# Supplementary material for: Pyruvate Dehydrogenase Kinase 1 inhibition mediated oxidative phosphorylation enhancement in cartilage promotes osteoarthritis progression
Source: BMC Musculoskelet Disord. 2023 Jul 20;24:597. doi: 10.1186/s12891-023-06585-6 (PMC10357736; doi:10.1186/s12891-023-06585-6)
Supplement: Supplementary file 1 — Additional file 1. [file 12891_2023_6585_MOESM1_ESM.zip › KEGG.Gsea.1653622432464/KEGG_BASAL_TRANSCRIPTION_FACTORS.html]

Details for gene set KEGG\_BASAL\_TRANSCRIPTION\_FACTORS[GSEA]

|  || Dataset | 55457\_collapsed\_to\_symbols.cls.cls #OA\_versus\_normal.cls.cls #OA\_versus\_normal\_repos |
| Phenotype | cls.cls#OA\_versus\_normal\_repos |
| Upregulated in class | normal |
| GeneSet | KEGG\_BASAL\_TRANSCRIPTION\_FACTORS |
| Enrichment Score (ES) | -0.30141106 |
| Normalized Enrichment Score (NES) | -1.0453914 |
| Nominal p-value | 0.3916084 |
| FDR q-value | 0.5777089 |
| FWER p-Value | 1.0 |
Table: GSEA Results Summary

  

Fig 1: Enrichment plot: KEGG\_BASAL\_TRANSCRIPTION\_FACTORS      
 Profile of the Running ES Score & Positions of GeneSet Members on the Rank Ordered List

  

| SYMBOL | TITLE | RANK IN GENE LIST | RANK METRIC SCORE | RUNNING ES | CORE ENRICHMENT || 1 | GTF2A2 | general transcription factor IIA subunit 2 [Source:HGNC Symbol;Acc:HGNC:4647] | 899 | 0.543 | -0.0188 | No |
| 2 | GTF2E1 | general transcription factor IIE subunit 1 [Source:HGNC Symbol;Acc:HGNC:4650] | 1108 | 0.494 | 0.0116 | No |
| 3 | GTF2E2 | general transcription factor IIE subunit 2 [Source:HGNC Symbol;Acc:HGNC:4651] | 1187 | 0.479 | 0.0506 | No |
| 4 | GTF2B | general transcription factor IIB [Source:HGNC Symbol;Acc:HGNC:4648] | 1315 | 0.455 | 0.0835 | No |
| 5 | GTF2A1 | general transcription factor IIA subunit 1 [Source:HGNC Symbol;Acc:HGNC:4646] | 1936 | 0.368 | 0.0700 | No |
| 6 | TAF9B | TATA-box binding protein associated factor 9b [Source:HGNC Symbol;Acc:HGNC:17306] | 2081 | 0.352 | 0.0920 | No |
| 7 | TAF7 | TATA-box binding protein associated factor 7 [Source:HGNC Symbol;Acc:HGNC:11541] | 3035 | 0.246 | 0.0411 | No |
| 8 | TBPL1 | TATA-box binding protein like 1 [Source:HGNC Symbol;Acc:HGNC:11589] | 3794 | 0.178 | -0.0011 | No |
| 9 | TAF12 | TATA-box binding protein associated factor 12 [Source:HGNC Symbol;Acc:HGNC:11545] | 3843 | 0.172 | 0.0113 | No |
| 10 | TAF6 | TATA-box binding protein associated factor 6 [Source:HGNC Symbol;Acc:HGNC:11540] | 4260 | 0.132 | -0.0086 | No |
| 11 | TAF7L | TATA-box binding protein associated factor 7 like [Source:HGNC Symbol;Acc:HGNC:11548] | 5955 | -0.002 | -0.1401 | No |
| 12 | GTF2H4 | general transcription factor IIH subunit 4 [Source:HGNC Symbol;Acc:HGNC:4658] | 6295 | -0.027 | -0.1639 | No |
| 13 | TAF9 | TATA-box binding protein associated factor 9 [Source:HGNC Symbol;Acc:HGNC:11542] | 6297 | -0.027 | -0.1614 | No |
| 14 | GTF2I | general transcription factor IIi [Source:HGNC Symbol;Acc:HGNC:4659] | 6588 | -0.049 | -0.1794 | No |
| 15 | TAF13 | TATA-box binding protein associated factor 13 [Source:HGNC Symbol;Acc:HGNC:11546] | 6900 | -0.072 | -0.1968 | No |
| 16 | TAF5 | TATA-box binding protein associated factor 5 [Source:HGNC Symbol;Acc:HGNC:11539] | 7594 | -0.127 | -0.2387 | No |
| 17 | TAF4B | TATA-box binding protein associated factor 4b [Source:HGNC Symbol;Acc:HGNC:11538] | 7596 | -0.127 | -0.2268 | No |
| 18 | TAF4 | TATA-box binding protein associated factor 4 [Source:HGNC Symbol;Acc:HGNC:11537] | 7966 | -0.153 | -0.2411 | No |
| 19 | GTF2A1L | general transcription factor IIA subunit 1 like [Source:HGNC Symbol;Acc:HGNC:30727] | 8548 | -0.197 | -0.2677 | No |
| 20 | TAF11 | TATA-box binding protein associated factor 11 [Source:HGNC Symbol;Acc:HGNC:11544] | 8860 | -0.222 | -0.2710 | Yes |
| 21 | GTF2F1 | general transcription factor IIF subunit 1 [Source:HGNC Symbol;Acc:HGNC:4652] | 8910 | -0.225 | -0.2536 | Yes |
| 22 | GTF2H1 | general transcription factor IIH subunit 1 [Source:HGNC Symbol;Acc:HGNC:4655] | 9455 | -0.269 | -0.2705 | Yes |
| 23 | GTF2IRD1 | GTF2I repeat domain containing 1 [Source:HGNC Symbol;Acc:HGNC:4661] | 9497 | -0.274 | -0.2480 | Yes |
| 24 | GTF2F2 | general transcription factor IIF subunit 2 [Source:HGNC Symbol;Acc:HGNC:4653] | 9876 | -0.309 | -0.2482 | Yes |
| 25 | STON1 | stonin 1 [Source:HGNC Symbol;Acc:HGNC:17003] | 9995 | -0.321 | -0.2272 | Yes |
| 26 | TAF5L | TATA-box binding protein associated factor 5 like [Source:HGNC Symbol;Acc:HGNC:17304] | 10906 | -0.418 | -0.2586 | Yes |
| 27 | TBP | TATA-box binding protein [Source:HGNC Symbol;Acc:HGNC:11588] | 11138 | -0.448 | -0.2344 | Yes |
| 28 | GTF2H3 | general transcription factor IIH subunit 3 [Source:HGNC Symbol;Acc:HGNC:4657] | 12001 | -0.593 | -0.2456 | Yes |
| 29 | TAF6L | TATA-box binding protein associated factor 6 like [Source:HGNC Symbol;Acc:HGNC:17305] | 12263 | -0.667 | -0.2032 | Yes |
| 30 | TAF2 | TATA-box binding protein associated factor 2 [Source:HGNC Symbol;Acc:HGNC:11536] | 12328 | -0.684 | -0.1437 | Yes |
| 31 | GTF2H2 | general transcription factor IIH subunit 2 [Source:HGNC Symbol;Acc:HGNC:4656] | 12612 | -0.806 | -0.0899 | Yes |
| 32 | TAF1 | TATA-box binding protein associated factor 1 [Source:HGNC Symbol;Acc:HGNC:11535] | 12881 | -1.192 | 0.0015 | Yes |
Table: GSEA details [plain text format]

  

Fig 2: KEGG\_BASAL\_TRANSCRIPTION\_FACTORS      
 Blue-Pink O' Gram in the Space of the Analyzed GeneSet

  

Fig 3: KEGG\_BASAL\_TRANSCRIPTION\_FACTORS: Random ES distribution      
 Gene set null distribution of ES for **KEGG\_BASAL\_TRANSCRIPTION\_FACTORS**
